# Origin of Oxygen in Graphene Oxide Revealed by ^17^O and ^18^O Isotopic Labeling

**DOI:** 10.1021/jacs.3c12543

**Published:** 2024-03-06

**Authors:** Christian E. Halbig, Bristy Mukherjee, Siegfried Eigler, Slaven Garaj

**Affiliations:** † Department of Chemistry, Biology and Pharmacy, Freie Universität Berlin, 14195 Berlin, Germany; ‡ Department of Materials Science and Engineering, National University of Singapore, 117575 Singapore, Singapore; § Department of Physics, Faculty of Science, National University of Singapore, 117551 Singapore, Singapore; ∥ Department of Biomedical Engineering, National University of Singapore, 117583 Singapore, Singapore

## Abstract

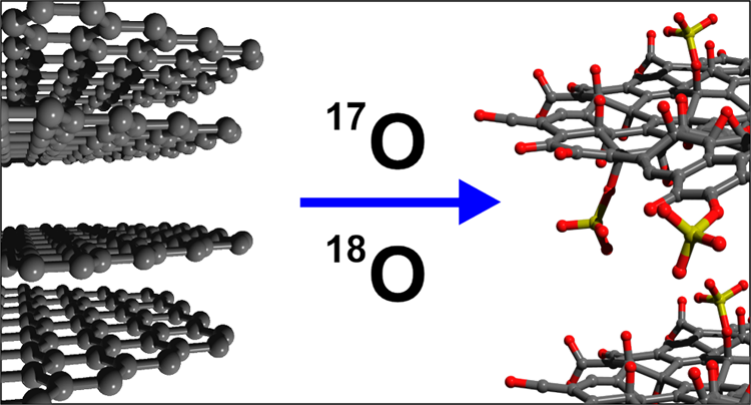

Wet-chemical oxidation of graphite in a mixture of sulfuric acid with a strong oxidizer, such as potassium permanganate, leads to the formation of graphene oxide with hydroxyl and epoxide groups as the major functional groups. Nevertheless, the reaction mechanism remains unclear and the source of oxygen is a subject of debate. It could theoretically originate from the oxidizer, water, or sulfuric acid. In this study, we employed 18O and 17O labeled reagents to experimentally elucidate the reaction mechanism and, thus, determine the origin of oxo-functional groups. Our findings reveal the multifaceted roles of sulfuric acid, acting as a dispersion medium, a dehydrating agent for potassium permanganate, and an intercalant. Additionally, it significantly acts as a source of oxygen next to manganese oxides. Through ^17^O solid-state magic-angle spinning (MAS) NMR experiments, we exclude water as a direct reaction partner during oxygenation. With labeling experiments, we conclude on mechanistic insights, which may be exploited for the synthesis of novel graphene derivatives.

## Introduction

Graphene oxide (GO) is a layered two-dimensional (2D) carbon material derived from graphene, with wide-ranging physical and chemical properties.^1^ Thus, GO has been the subject of intensive research and found applications in electronic devices (transistors, sensors, solar cells, batteries, etc.), biomedicine (molecular transporter, antimicrobial surface, biosensing, bioimaging, etc.), and nanofiltration.^2^

**Figure 1:**
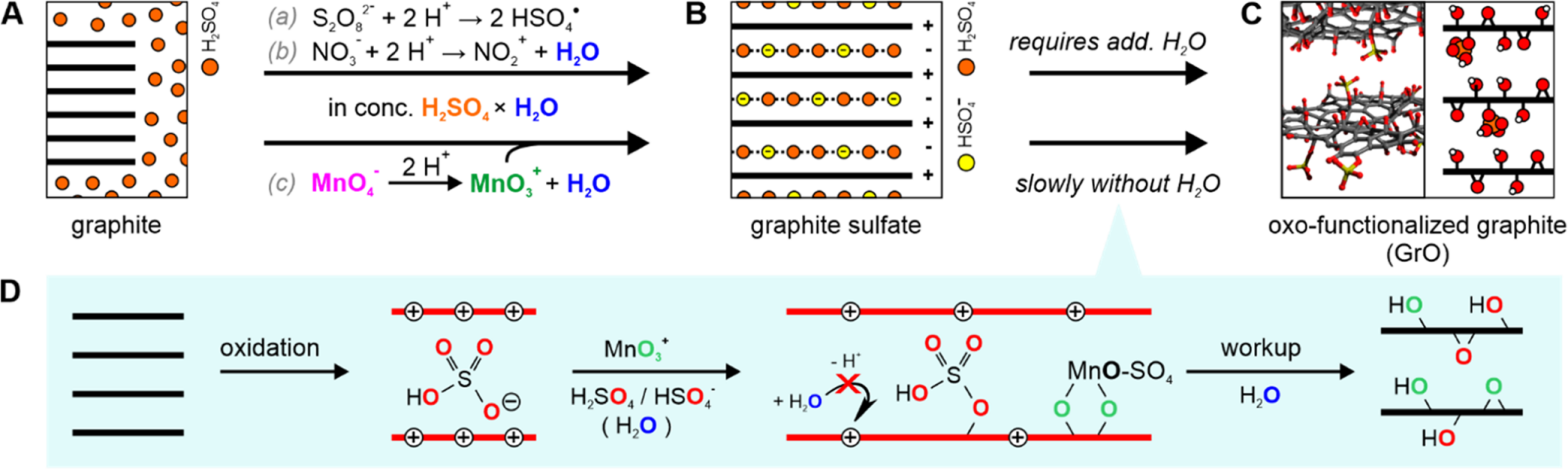
(A–C) Scheme illustrating the route from graphite to oxo-functionalized graphite (graphite oxide, GrO) via graphite sulfate. The dashed lines between the yellow and red colored spheres (H_2_SO_4_/HSO_4_^–^) in (B) illustrate the hydrogen-bond network in the guest layer.^4c^ The *in situ* formation of the reactive species using three different oxidation agents, namely, persulfate, nitrate, and permanganate, is shown above and below the reaction arrows (a–c). Using persulfate (a) and nitrate (b), the reaction stops with the formation of stage-1 graphite sulfate,^4a,4c^ but slowly in the presence of permanganyl cations to GrO even without further addition of water (c). (D) Scheme illustrating the mechanism of the formation of oxo-functional groups on the graphene sheets within a graphite crystal. The origin of oxygen in the functional groups was traced with isotopically labeled reagents. In the presence of permanganyl cations, the graphite intercalation compound (GIC) is converted to highly functionalized GO (∼65%) upon aqueous workup with a majority of oxo-functional groups stemming from sulfuric acid and permanganate.

Most popular ways to prepare GO based on wet-chemical oxidation of bulk graphite under subsequent aqueous workup have been elaborated up to now.^1a,3^ All of these methods have in common that graphite is stirred in concentrated sulfuric acid and/or nitric acid with sufficiently strong oxidizers like nitrate, persulfate (PS), chlorate, or permanganate (PM). Subsequent aqueous workup leads to the final oxygenated product (Figure 1A–C), which can be further exfoliated to single sheets of GO, for instance, by ultrasound treatment.^1b,1c,4^ These processes lead to functionalization of each carbon layer in a graphite crystal with oxo-functional groups. According to the widely accepted structural model of Lerf and Klinowski, materials prepared using potassium permanganate (PM) in concentrated sulfuric acid (cf. method of Hummers and Offeman) typically consist of a functionalized hexagonal carbon framework, where approximately ^2^/_3_ of all sp^2^ carbon atoms are decorated with epoxy, hydroxy, and organosulfate groups, whereas minor amounts of carboxy, carbonyl, phenolic hydroxy groups, and negligible traces of other oxygen-containing structural motifs can be present at defect sites and the rims of a GO particles.^1c,5^ As shown by 2D ^13^C 3Q/SQ correlation solid-state NMR spectra and ab initio modeling, highly functionalized domains with hydroxyl groups vicinal to epoxide groups coexist next to unfunctionalized sp^2^ domains.^5c,5f,6^ However, the ultimate degree of functionalization, composition of functional groups, and quality of the carbon lattice depend strongly on the constitution of the initially used graphite, the chosen oxidizer, and reaction parameter for oxidation and subsequent workup or purification, respectively.^1b–1d,2e,5e,7^

From a mechanistic point of view, oxidation and intercalation of graphite in concentrated sulfuric acid using salts of nitrate (N), persulfate (PS), and potassium permanganate (PM) was well studied: electron transfer from graphite to the oxidation agent results in the intercalation of hydrogen sulfate and sulfuric acid for charge compensation and eventually to the formation of graphite sulfate—a stable and blue stage-1 graphite intercalation compound (GIC) with an idealized formula of [C_24_^+^ * HSO_4_^–^ * 2 H_2_SO_4_]*_n_* (Figure 1B).^4b,4c^ Due to the intercalation, the interlayer distance between the positively doped graphene layers is increased from initially 3.35–7.98 Å.^4b^ Using N and PS, the reaction stops with the formation of graphite sulfate, but after aqueous workup and subsequent exfoliation, small amounts of GO with a degree of functionalization of about 4% and a highly intact carbon lattice can be obtained.^4a,4c^ In contrast, GICs prepared with PM are not stable in the reaction mixture but the intermediary formed intercalates become slowly functionalized even without further addition of water.^8^ After analog aqueous workup, GO with a higher degree of functionalization, typically in the range of 40–70%, can be obtained in high yields.^9^ However, the fundamental carbon lattice frequently suffers from partial degradation due to so-called overoxidation.^5e,10^

Especially the oxidation of graphite with potassium permanganate is of high importance due to its simplicity and high yield of single-layer GO; however, the fundamental mechanism is yet not clear and only indirect evidence has been given by other reports.^7b,11^ It is a general consensus that MnO_3_^+^-related species are involved in the oxidation.^8,11,12^ For instance, Huang et al. and Li et al. investigated the formation and hydrolysis of cyclic permanganate ester on the carbon lattice of graphite, which many researchers believe to be formed analogously to the *syn*-dihydroxylation of olefins.^11b^ In another study, Kang et al. identified a second oxidation step during aqueous workup of oxidized graphite in the reaction mixture of sulfuric acid with potassium permanganate.^7b^ In contrast, Dimiev et al. recently proposed that water in the reaction mixture is the main source of oxygen, and manganese species serve only to provide sufficiently strong redox potential for the observed oxygenation.^8^ Other groups have suggested that ozone, formed during the decomposition of manganese species in sulfuric acid, could be a plausible source.^13^ Morimoto et al. critically questioned some of the aforementioned conclusions and, among other findings, ruled out water and ozone as reactants by experiments with ^18^O-labeled water.^12^ However, a definitive answer was not found yet as all of these results are either based on computational studies or indirect observations.^7,8,11,13b^

Here, we applied different combinations of ^17^O- and ^18^O-labeled reagents to elucidate the source of oxo-functional groups in GO by thermogravimetric analysis coupled with mass spectrometry (TGA-MS) and ^17^O solid-state nuclear magnetic resonance spectroscopy (ssNMR).^14^ We observed that intercalated sulfuric acid is not only essential as an intercalant and dehydration agent but also an important source of oxygen. In contradiction to prior studies, we found evidence that oxygen of water, either present in the reaction mixture as a part of concentrated sulfuric acid or added later during aqueous workup, is not introduced in oxo-functional groups (Figure 1D) of GO.

## Results and Discussion

### Oxidative Intercalation Experiments

GIC formation is the initial and crucial step for GO formation, accompanied by the increase of interlayer distance of graphene layers due to penetration of sulfuric acid and hydrogen sulfate between the induvial graphene layers.^4a,4c^ Therefore, we monitored the formation and stability of three different GICs (GIC^PS^, GIC^N^, GIC^PM^), prepared under controlled environmental conditions excluding moisture (glovebox or overlaid with *n*-hexane, Figure S1), using either ammonium persulfate (PS), sodium nitrate (N), or potassium permanganate (PM) from day 1 to 7 by Raman spectroscopy.

**Figure 2:**
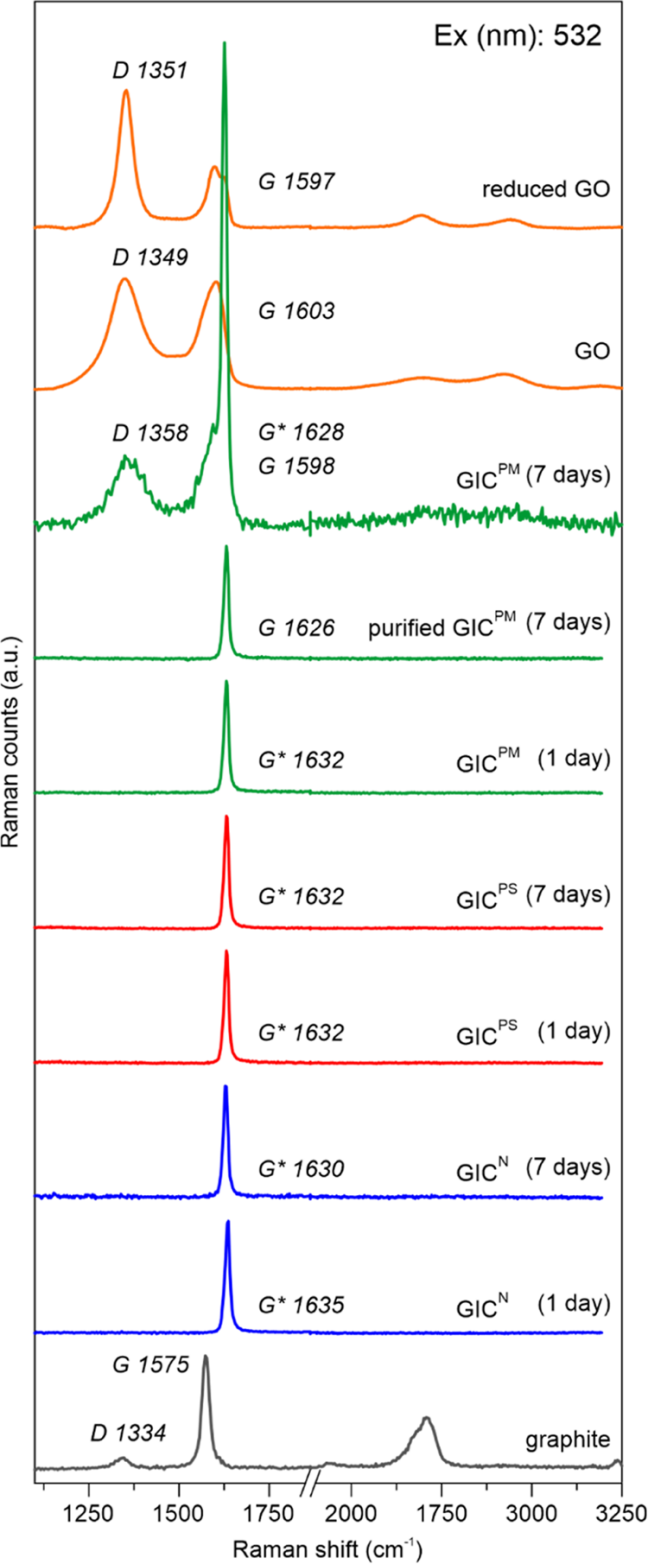
Raman spectra of graphite and the thereout-prepared GICs by using sodium nitrate (GIC^N^, blue), sodium persulfate (GIC^PS^, red), and potassium permanganate (GIC^PM^, green) after 1 and 7 days in the reaction mixture. GIC^PM^ (purified) was washed after 2 h reaction time with pure sulfuric acid and stored in the same for 7 days. Spectra of GO and reduced GO (graphene) are shown at the most top in orange. The *D*, *G*, and G* modes (asterisk indicates blue shift) are annotated with italic letters together with their corresponding Raman shift. All spectra were normalized to the *G* or G* peak, respectively.

All three oxidizers convert graphite to stage-1 GICs, within a few minutes (GIC^PM^) or several hours (GIC^PS^, GIC^N^). The related Raman spectra indicate doping by the blue shift of the *G* modes from 1575 to 1626–1635 cm^–1^ (G*, Figure 2).^4a–4d^ After 7 days of reaction time, GIC^PS^ and GIC^N^ remained stable in the reaction mixture, and no differences in the Raman spectra could be observed. In contrast, the reaction of graphite with PM did not stop with GIC^PM^ formation but proceeded slowly, as indicated by the appearance of the D band (∼1358 cm^–1^) and the additional *G* band (∼1598 cm^–1^). These new spectral features had similar line shapes as highly functionalized GO, and overlapped with signals from residual stage-1 intercalated layers (Figures 2 and S2). We were able to suppress this reaction by removing excess oxidizer via repetitive centrifugation, as shown in the Supporting Information (Figure S1B). These observations indicate that intercalation proceeds quickly, and the presence of additional oxidant further drives slow functionalization of the carbon lattices.

For GIC^N^ and GIC^PS^, the redox potentials for *in situ* formed nitronium cations (2NO_2_^+^ + 2e^–^ → N_2_O_4_*E*_0_ = 1.5 V vs NHE) or sulfate radicals (SO_4_^•–^ + e^–^ → SO_4_^2–^*E*_0_ = 2.6 V vs NHE) are only sufficient to *p*-dope graphite and facilitate intercalation,^15^ but a further functionalization of the carbon lattice is only observed after aqueous workup, as shown in our earlier studies, resulting in material with a typical degree of functionalization of 4%.^4a,4c^ Manganese oxides are also known to be strong oxidizers, but in contrast to the aforementioned, they are also known to be versatile oxygen donors (cf. *syn*-dihydroxylation of olefins).^14c,16^ This is reflected by the fact that oxidation of graphite with PM (cf. method of Hummers and Offeman) typically leads to GO with a degree of functionalization of more than 50%.^9^

However, the exact point when the majority are formed is not clear as Raman spectroscopy is able to provide information about the degree of functionalization of multilayer GO or graphite oxide, respectively, as the line shape of materials with 4 and 50% functionalization look similar.^17^ It seems plausible that for now only minor amounts of functional groups are formed as G* is still present in the Raman spectra after 7 days of oxidation, and a full conversion to GrO is only observed upon water addition in the presence of manganese oxides (Figures S3 and S4). Whether a slow [2 + 3] cycloaddition of the oxidant, or reaction with *in situ* formed oxygen traces, due to the degradation of MnO_3_^+^ in the acid mixture, is responsible for the functionalization cannot be clarified at this point,^13a^ but since PM alone is sufficient to convert graphite to graphite sulfate, even faster than N and PS, the addition of these as co-oxidants is unnecessary for the synthesis of GO.

### Isotopic Labeling

To directly identify the oxygen-donating reactant, first, the reference material was prepared using unlabeled sulfuric acid (16A) and water (16W) as reference (GO-16A-16W) material. Second, ^18^O-labeled sulfuric acid was used and normal water was used for workup, resulting in GO-18A-16W. Third, normal sulfuric acid (16A) and ^18^O-labeled water were used for workup to form GO-16A-18W.

The reference material GO-16A-16W had a degree of functionalization of about 59% and is decorated with mainly epoxide, hydroxyl on the surface, and some carbonyl groups at the rims and defect sites, as evidenced by X-ray photoelectron spectroscopy (XPS) and ^13^C solid-state nuclear resonance spectroscopy (^13^C ssNMR, Figure S5).^5a,5b,5f^ Minor amounts of organosulfates are present as well, as indicated by the detection of ∼5% sulfur in the combustion elemental analysis (EA) and two signals with binding energies of ∼232 eV (S 2s) and ∼168 eV (S 2p) in the XPS survey spectra (Figure S5C).

**Figure 3:**
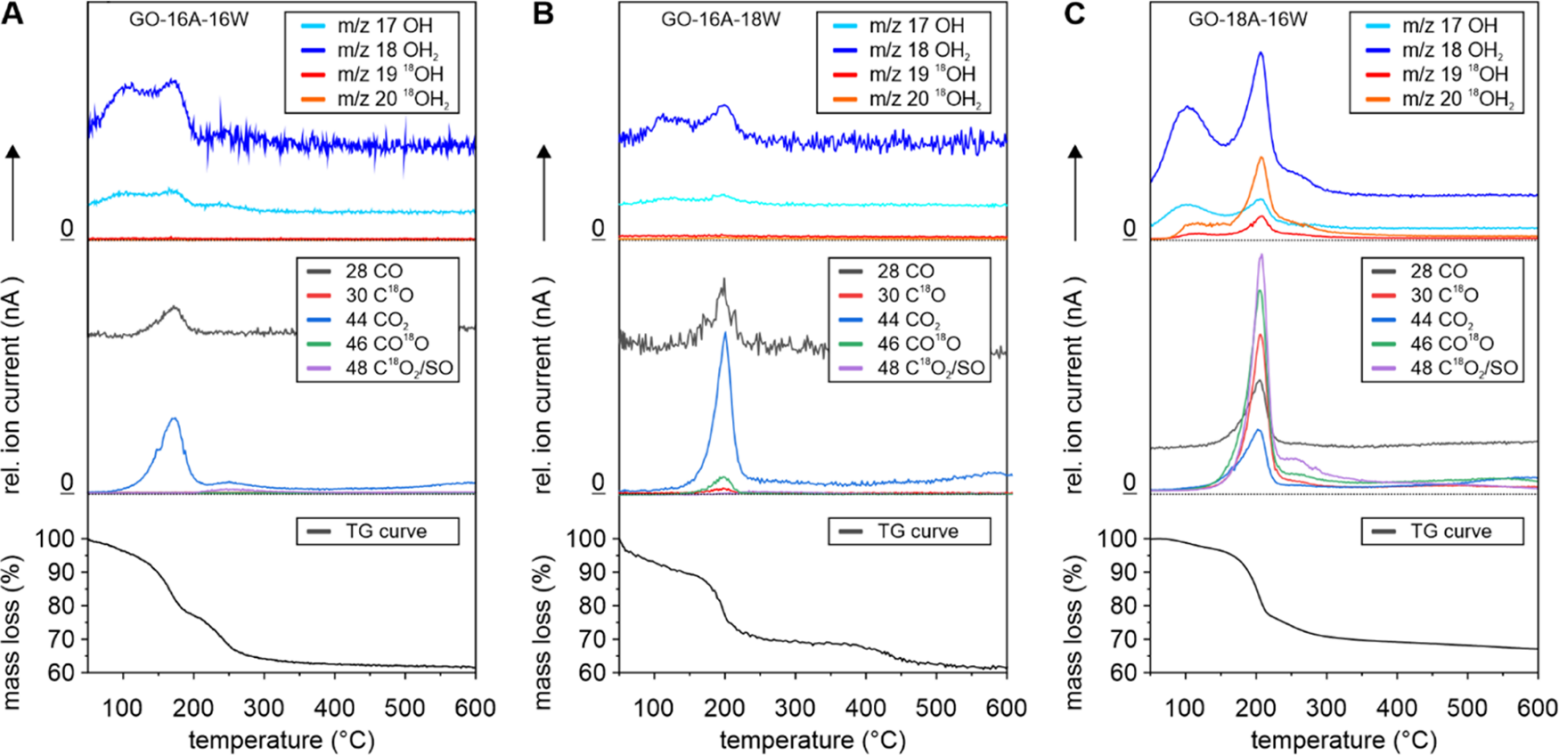
(A–C) Ion traces from TGA-MS analysis of three different GO samples prepared with potassium permanganate. (A) Synthesis of GO-16A-16W was conducted with regular sulfuric acid, followed by aqueous workup with regular deionized (DI) water. (B) The material GO-16A-18W was obtained in a similar way, but ^18^O-labeled water was used for aqueous workup instead, while (C) ^18^O_4_^–^ sulfuric acid was used to synthesize GO-18A-16W.

Through thermogravimetric analysis coupled with mass spectrometry (TGA-MS), we can identify three main steps of decomposition (Figures 3A and S5B). In the first step up to ∼120 °C, physisorbed water was released, indicated by mass traces with *m*/*z*  17 (OH) and 18 (OH_2_). The majority of functional groups, namely, epoxide and hydroxyl groups, decomposed between ∼120 and ∼200 °C, resulting in a second peak of fragments with *m*/*z*  17 and 18, along with additional traces related to the formation of carbon monoxide (*m*/*z* 28) and carbon dioxide with *m*/*z* 44, as a result of the degradation of the carbon lattice. In the third region between 200 and 300 °C, organosulfate groups decompose and additional traces for SO_2_ with *m*/*z*  64 could be detected.^5b^

**Figure 4:**
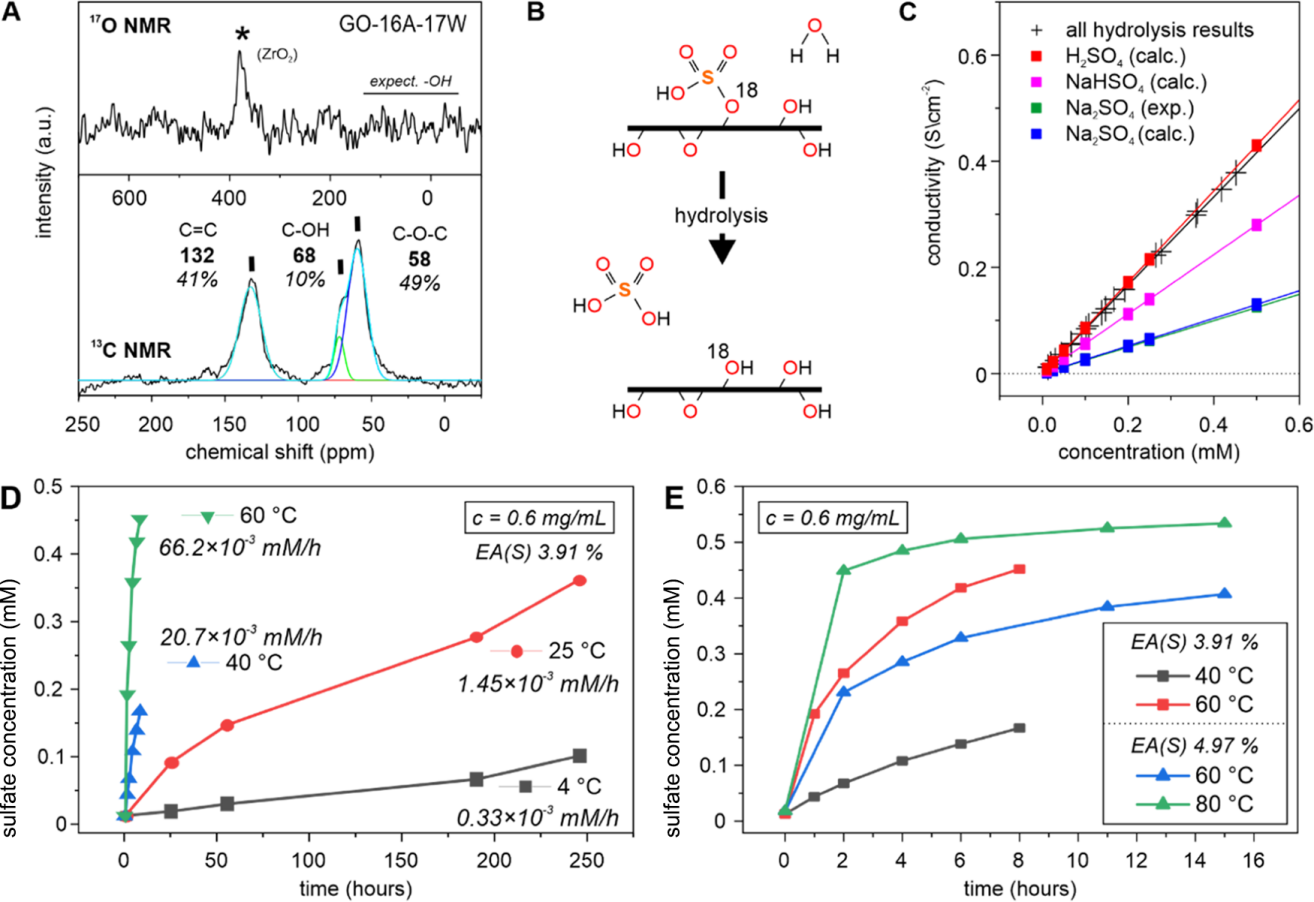
(A) ^17^O (top) and ^13^C solid-state NMR spectra (bottom) for GO prepared with ^17^O-water in the reaction mixture (GO-16A-17W). The signal with the asterisk represents a background signal from the used zirconia rotor, whereas the black line indicates the region where hydroxyl groups can be expected. The related chemical shifts are shown in bolt numbers together with the integrated areas in percent. (B) Scheme illustrating the hydrolysis of organosulfates on GO. (C–E) An aqueous dispersion GO at a concentration of 0.5 mg/mL was incubated at different temperatures (4, 25, 40, 60 °C). The conductivities and the free sulfate concentration of the aqueous solution were measured after removal of GO by repetitive centrifugation. (C) Plot of the conductivity of the solution versus the determined concentration of sulfate via ion chromatography. For comparison, the experimental (expt) and theoretical (calcd) values of sodium sulfate and sodium hydrogen sulfate are shown additionally. The experimental data points (black crosses) match best with the theoretical conductivity values for sulfuric acid. (D, E) Increase of free sulfate over different time scales at different temperatures.

A replacement of regular water with ^18^OH_2_ during aqueous workup (GO-16A-18W) led to the emergence of negligible signals for heavier ^18^O-containing fragments with *m*/*z* 19 and 20, and weak signals for *m*/*z* 30 and 46 (Figure 3B). The same weak signals were previously detected by Morimoto et al. in an experiment to study a possible functionalization of the carbon lattice and *in situ* formed ozone traces.^12^ However, freeze-dried GO-16A-16W samples, which were redispersed in ^18^O-water or D_2_O for 1 day, and subsequently freeze-dried again led to same results (Figure S6). This indicates that the origin of these signals could also stem from remnant traces of adsorbed water or minor covalently bound ^18^O-containing functional groups due to possible partial hydrolysis of organosulfates (Figure 4B).

Further evidence for a nonactive role of water during GO synthesis was obtained by diluting 97.5% sulfuric acid with ^17^O (90%) to a final concentration of 95% before the oxidizer was added to the graphite. Consequently, roughly half of the water molecules present in the acid were ^17^O-labeled (Table 1). After aqueous workup, we conducted ^13^C and ^17^O solid-state NMR (ssNMR). While well-resolved ^13^C ssNMR spectra with unambiguous signals for epoxide (∼60 ppm), hydroxyl (∼70 ppm), and unsaturated C–C bonds (∼132 ppm) could be obtained, no relevant ^17^O signals were detectable. Instead, only a small signal from the zirconia rotor was present at about 380 ppm (Figure 4A), outside the expected region below 100 ppm, e.g., around 45 ppm reported for hydroxyl groups.^14a,14b^ Beyond, this finding also excludes a significant functionalization of the graphene lattice by ozone, as proposed earlier elsewhere.^13,18^

**Table 1:** Exemplary Amounts of Substances Calculated for a Microscale Oxidation with 3 wt Equiv of Oxidizer with Respect to Carbon^a^

substance	*v* [mL]	*m* [mg)	*n* [mmol]	mol. ratio
H_2_SO_4_ (97.5 wt %)	4	∼7340	72.97	**8.7**
→ H_2_O content			10.19	**1.2**
H_2_SO_4_ (95 wt %)			71.10	**8.5**
→ H_2_O content			20.37	**2.4**
carbon		100	8.33	**1**
KMnO_4_		300	1.90	**0.2**
→ condensed H_2_O		34.20	1.90	**0.2**
total water content in 97.5 wt % H_2_SO_4_		217.7	12.08	**1.45**
total water content in 95 wt % H_2_SO_4_		401.2	22.27	**2.67**

a Most of the water stems from concentrated sulfuric acid itself, only minor traces of water are formed by dehydration of permanganate. The ratios of the used substances are representative of a typical formulation according to Hummers and Offeman.^1a^ The numbers refer to regular compounds (^16^O), and no isotopic labeling was considered.

Most interestingly, when ^18^O_4_-sulfuric acid was used to prepare GO-18A-16W, intense ion traces for ^18^O-containing fragments appear between 50 and 300 °C, next to signals for ^16^O-related fragments (Figure 3C). Ion traces of ^18^OH_n_ fragments (*m*/*z*  19 and OH_2_: *m*/*z*  20) in the temperature range between 150 and 230° have an intensity of about ∼60% with respect to their unlabeled counterparts, while the intensity of C^18^O with respect to C^16^O is about 200%. The shoulders visible for signals with *m*/*z*  30, 44, 46, and 48 at temperatures higher than ∼230 °C originate from the decomposition of organosulfate groups (SO_2_), as evidenced by the presence of an ion trace with *m*/*z*  64.^5b^ Such intense signals were counterintuitive as sulfuric acid was expected to solely act as a dehydration agent and intercalant. By the design of the experiment, and as water was excluded as a reaction partner, other ^16^O-containing fragments should exclusively stem from permanganate. A possible transfer of oxygen isotopes between sulfuric acid and manganese oxides in the reaction mixture could be excluded by a series of ^17^O liquid NMR reference experiments in the absence of graphite. While an oxygen transfer between water and concentrated sulfuric acid can be clearly seen within a short time after the addition of ^17^O-water to unlabeled sulfuric acid, at no time, ^17^O signals for MnO_4_^–^ arise (Figure S7).^19^

### The Role of the Components

Based on the observation that water does not significantly contribute to oxygenation but strongly influences the reaction rate and that oxygen from sulfuric acid is somehow transferred to the carbon lattice, the role of all components in the reaction mixture has to be discussed in a new context.

During oxidation of olefins like oleic acid, the majority of oxygen is donated from the permanganate anion after [3 + 2] cycloaddition and hydrolysis in neutral to strongly alkaline solutions;^14c^ however, oxidation of graphite is conducted in concentrated sulfuric acid. The presence of free MnO^3+^ and its adduct MnO_3_–OSO_3_ in concentrated sulfuric was discussed in detail earlier, and especially the latter could be the actual species involved in the formation of cyclic intermediates on the graphene lattices in graphite.^20^ Theoretical studies predicted that manganese oxides of the type MnO_3_L (e.g., MnO_3_Cl) can undergo [3 + 2] cycloadditions on unsaturated compounds.^21^

Huang et al. studied the hydrolysis of eventually formed cyclic intermediates by density functional theory calculations and indicated that C–O bond cleavage during hydrolysis is energetically favored over the Mn–O bond, implying that oxygen from water is incorporated. However, our TGA-MS and NMR data contradict since water molecules are barely incorporated in GO.^11a,12^ Considering for sample GO-18A-16W that permanganate is completely dehydrated to MnO_3_^+^, the content of ^16^O-water in the reaction mixture before aqueous workup would be just 14%—too little to explain the observed strong ^16^O-related ion traces in the TGA-MS spectra (Figure 3C). Hence, a double oxygen transfer from manganese species is thus more plausible and favored in accordance with the widely accepted mechanism of dihydroxylation.^12,14c^ The only other possibility would be the formation of oxo-functional groups by unimolecular decomposition of intercalated Mn–VII species,^13a^ but the reaction with graphite was found to be very slow (Figure 1), and the addition of water during aqueous workup would favor four- and six-electron oxidation of water to ozone—a reaction that does not occur significantly with graphite.^12^

It is also reasonable that other ^18^O-related signals in our TGA-MS spectra for epoxide and hydroxyl group signals can only originate from sulfuric acid. It is known that certain olefins do not only polymerize but also form organosulfates and alcohols in the presence of sulfuric acid in a wide range of concentrations.^22^ Here, the formation of organosulfates is favored with increasing acid concentration due to the increasing deactivation of water by protonation.^22a^

The situation is similar for the functionalization of graphite or graphene, respectively, but not exactly the same due to the presence of an enlarged sp^2^ carbon network. Most importantly, the water content in the reaction mixture determines which reactions take place and defines their reaction rates. Lankshear and Royer previously noted that the chemical stability of permanganyl cations increases with water content up to a certain threshold, and that other manganese species are formed in anhydrous oleum that appear to be unsuitable for the conversion of graphite into graphite sulfate and GO, respectively.^8,20^

In contrast to olefins, the cationic intermediate is not formed by protonation but by oxidation and is stable for long periods of time in highly concentrated sulfuric acid (Figure 2). On the other hand, not only is the positive charge distributed over 24 carbon atoms but also the negative charge in the guest layer is delocalized by the Grotthuß mechanism.^4c^ Therefore, hydrogen sulfate cannot sufficiently interact with the delocalized positive charge to form a covalent bond. Addition of water would lead not only to a destabilization of the hydrogen-bond network between the confined sulfur species between the graphene sheets but also to an increase of hydrogen sulfate and sulfate.

From earlier conducted ab initio molecular dynamics simulations, we also know that oxo-functional groups in varying densities can be stable on a p-doped graphene lattice in the presence of concentrated sulfuric acid.^4c^ Here, the charge density was always two positive charges per 60 carbon atoms, which roughly matches the charge density in graphite sulfate. Thus, at an ideal concentration of water, MnO_3_^+^ could not only oxidize the carbon lattice and donate a certain number of oxo-functional groups but also facilitate bond-formation between intercalated sulfur species and the p-doped carbon lattice and eventually form organosulfate groups (Figure 1D). In the case of labeled sulfuric acid, the structure matches R_3_C–^18^O-S^18^O_3_H. These organosulfates on the graphene lattice are instable, especially when their density is high,^5b,23^ and easily hydrolyze to hydroxyl groups and free sulfuric acid (Figure 4B,C). We had a look at the kinetics of hydrolysis of this functional group by preparing several freshly prepared GO dispersions with a specific concentration and observed that the reaction rate strongly correlates with the temperature (Figure 4C,D). These results are in agreement with observations for sulfation of olefins and their hydrolysis behavior.^22^ In contrast, esterification of ^16^O-hydroxyl groups stemming from permanganate and subsequent hydrolysis would not lead to R-^18^OH groups.

Ultimately, hydroxyl groups formed on the carbon lattice by the acid component and the oxidizer PM will dehydrate to epoxide groups with pronounced stability,^5f^ especially where sterically permitted and the resulting structure is more stable. Additionally, the possibility of a migration and clustering of functional groups to dominantly highly functionalized sp^3^ domains on and low functionalized sp^2^ domains has been frequently raised and witnessed both theoretically and experimentally.^2h,5c,6,12,24^ Both processes can explain the presence of a dominant major structural pattern, namely, vicinal alcohol groups next to epoxide groups on graphene oxide.^5f^

## Conclusions

The wet-chemical oxidation of pristine graphite to graphite oxide and graphene oxide, respectively, should be understood as a two-step reaction: a quick oxidative p-doping reaction, resulting in GIC formation, and a slower oxygenation reaction by permanganate species, resulting in covalent bond-formation (Figure 2D). Thereby, sulfuric acid and the oxidizer permanganate are both the two major oxygen-transferring reactants. By TGA-MS and ^17^O ssNMR, we provided evidence that water is not directly involved in the formation of oxo-functional groups but may enhance the stability of permanganyl cations, which possess a high oxidation potential, and this facilitates efficient formation of graphene oxide. Understanding the role of each component helps not only to optimize the reaction conditions but also to identify other useful reactants for targeted synthesis of novel materials, for example, by selecting different acid mixtures and nucleophiles capable of binding or transferring functional groups to p-doped graphene layers.

## Supporting Information Available

Preparative techniques; experimental data; exclusion of atmospheric oxygen and humidity (Figure S1); Raman spectrum of GO and GIC^PM^ after 7 days (Figure S2); photograph of oxidized graphite with permanganate before and after addition of water and hydrogen peroxide (Figure S3); Raman spectra and Raman maps of GIC^PM^ after 7 days of oxidation (Figure S4); ^13^C solid-state NMR spectra, TGA-MS and XPS spectra of GO-16A-16W and GO-18A-16W (Figure S5); TGA-MS and FT-IR data of GO, incubated with H_2_O, ^18^O–H_2_O, and D_2_O (Figure S6); ^17^O NMR spectra of sulfuric acid, sulfuric acid with ^17^O-water, and recovered KMnO_4_ from ^17^O-labeled concentrated sulfuric acid (PDF)
