# Supplementary material for: Origin of Oxygen in Graphene Oxide Revealed by 17O and 18O Isotopic Labeling
Source: J Am Chem Soc. 2024 Mar 6;146(11):7431–8. doi: 10.1021/jacs.3c12543 (PMC10958498; doi:10.1021/jacs.3c12543)
Supplement: Supplementary file 1 — ja3c12543_si_001.pdf [file ja3c12543_si_001.pdf]

Origin of oxygen in graphene oxide revealed by ^{17}O and ^{18}O isotopic labelling

Christian E. Halbig*[a, c], Bristy Mukherjee[b], Siegfried Eigler[a], Slaven Garaj*[b,c,d]

a) Department of Chemistry, Biology and Pharmacy, Freie Universität Berlin, 14195 Berlin, Germany.
b) Department of Materials Science and Engineering, National University of Singapore, 117575 Singapore, Singapore.
c) Department of Physics, Faculty of Science, National University of Singapore, 117551 Singapore, Singapore.
d) Department of Biomedical Engineering, National University of Singapore, 117583 Singapore, Singapore.
* *Corresponding authors.*

KEYWORDS: graphene, graphene oxide, isotopic labelling, ^{17}O & ^{13}C solid state NMR.

SUPPLEMENTARY INFORMATION

Experimental methods and synthetic Procedures

Thermogravimetric analysis coupled with mass spectrometry (TGA-MS): Netzsch Jupiter F3 STA, Aëolus III QMS with N5 helium as a carrier gas. Solid state nuclear magnetic resonance spectrometry (ssNMR): Bruker AVNEO400, 3.2mm probe head, 8 kHz spinning frequency, single pulse mode, 10 seconds T1 time for ^{13}C and 5 seconds T1 time for ^{17}O nuclei. ^{17}O liquid NMR: Jeol ECP500, 67.8 Mhz, 10 seconds T1, solvent where applicable: D_2O. Raman spectroscopy: WITec alpha300 confocal Raman spectrometer, 532 nm laser. X-ray photoelectron spectroscopy (XPS): Specs EnviroESCA, K_α = 1486.71 eV. Elemental Analysis (EA): ThermoFisher Scientific FlashSmart CHNS Elemental Analyser. Fourier-Transform infrared spectrometer (FT-IR): Bruker Alpha II with Diamon-ATR. Ion Chromatography (IC): Metrohm 930 Compact IC Flex equipped with a Metrohm A-supp 5 column (15cm x 4mm diameter) and a conductivity detector.

Chemicals were commercially purchased and used as obtained. Sigma-Aldrich (USA): ^{18}O-water (> 98%); sulfuric acid (97.5%); potassium permanganate (99%), trifluoro acetic acid (99%), hydrogen iodine (55%, no stabilizer). Cortecnet (France): ^{17}O-water (>90% and 40%); ^{18}O-water (> 98%); $^{18}O_4$-sulfuric acid in $^{18}OH_2$ (96 %, ^{18}O-enrichment grade for both: 96%); Sigma-Aldrich/Merck (Germany): D_2O (99%); Asbury Carbon Mills (USA): Natural graphite grade 3061.

General procedure of GO preparation: Graphite (1 g) was stirred in sulfuric acid (40 mL; 97.5%). Meanwhile, three mass equivalents of $KMnO_4$ (3 g) were added in small portions to the mixture within two hours. Subsequently, we slowly added in consecutive order sulfuric acid (60 mL; 20%; 4 h), water (100 mL; 16 h) and finally hydrogen peroxide (40 mL; 5%, 20 min). The liquids were used out of the fridge (4 °C) and the reaction mixture was additionally cooled by an external refrigeration coil (4 °C). The resulting oxygenated graphite (GrO) was washed by repetitive centrifugation with water for at least 6 times until pH is near neutral. Exfoliation of GrO to GO was achieved by two minutes of mild tip sonication (~ 10-15 W). Big particles were removed from the dispersion by centrifugation at low speed (2000 RCF, 3 min, 3 times). Smallest particles in the dispersion were finally removed by a single run at high speed (12000 RCF; 40 min) and discarding of the supernatant. The final concentration was determined by freeze-drying an aliquot of the obtained dispersion. The obtained material was termed GO-16A-18W as no isotopic labeled reagents were used. EA: C = 38.9 %; H = 2.52 %; N < 0.5%; S = 4.97 %. ^{13}C ssNMR (600 MHz, **Figure S5A**): δ 128.7 (C=C, 40 %), 68.4 (C-OH, 26 %), 57.1 (Epoxide, 34 %). TG (**Figure S5B**): -39.42 % (50°C - 600 °C).

Isotopically labelled GO: The general procedure for GO preparation, as outlined above, was downscaled using 50 mg graphite. In one reaction, $^{18}O_4$-labelled sulfuric was used and worked up with regular DI water (GO-18A-16W). In another reaction, regular sulfuric acid was used, but the reaction was worked up with ^{18}O-labelled water (GO-16A-18W). A total amount of 4 mL ^{18}O-labelled water was used to wash the sample for four times. The obtained material was freeze-dried and used for TGA-MS.

Isotopic labelled ^{17}GO with diluted H_2SO_4: The reaction follows the general procedure but with different quantities: Graphite (150 mg), sulfuric acid (4.5 mL / 97.5%); $KMnO_4$ (3 wt. eq. = 450 mg). In this experiment, the initial sulfuric acid was diluted with ^{17}O-water (70%) to a concentration of 95% before graphite and permanganate were added. The obtained material was termed GO-16A-17O. EA: C = 38.9 %; H = 2.52 %; N < 0.5%; S = 4.97 %. ^{13}C ssNMR (600 MHz, **Figure 4A**): δ 132.3 (C=C, 37.0 %), 68.1 (C-OH, 22 %), 58.0 (Epoxide, 41 %).

Reduction of GO: 300 nm SiO_2/Si wafer cleaned with piranha solution for at least 4 h and subsequently rinsed with MilliQ. water. Then, a small amount of aqueous GO dispersion was diluted 1:1 with triple distilled methanol and either drop casted, or deposited by Langmuir-Blodgett technique on a freshly cleaned wafer [1]. The coating was dried under vacuum for at least 1 h. Then, the wafer was placed in a glass chamber, reduced for 10 min by vapor of hydrogen iodine and trifluoro acetic acid (equal in volumes, vapor generate on hotplate at 80 °C), and finally rinsed with MilliQ water.

Preparation of graphite intercalation compounds (GICs) for Raman analysis: The preparation of GICs was conducted under inert gas condition in an Argon filled glove box (H_2O and O_2 < 0.01% ppm). Graphite (50 mg) and sulfuric acid (5 mL, 97.5%) were mixed in a small glass vial at room temperature with sodium persulfate, sodium nitrate or potassium permanganate and continuously stirred at room temperature. A maximum of one weight equivalent of oxidizer with respect to graphite was added each day at once, with a maximum of 3 weight equivalents. For analysis, some GIC^{PS} and GIC^{N} crystals were transferred together with a small amount of sulfuric acid on a glass slide. A cover slip was placed on top and common adhesive tape was used to seal the sample (**Figure S1A**).

Due to the intense color of the reaction mixture containing potassium permanganate, GIC^{PM} particles were centrifuged for few for 10 times outside of the glove box. After each run, the supernatant was removed and replaced with fresh sulfuric acid until the mixture was colorless. To protect the GICs from ambient humidity during washing we added a certain volume *n*-hexane (**Figure S1B**). Obtained purified GIC^{PM} crystals were transferred in the glove box on glass slides as aforementioned. For reference, one sample of graphite ("washed GIC^{PM}") was oxidized with only one weight equivalent of PM for two hours. It was washed with pure sulfuric acid by the above outlined procedure.

Exchange reactions with GO in H_2O, D_2O and ^{18}O-water: About 3 mg freeze dried GO-16A-16W was redispersed in approximately 3 mL DI water, D_2O (99%), or ^{18}O-labelled water. After 24 h, the material was freeze dried and analyzed by TGA-MS and FTIR-analysis.

Exchange reactions between H_2O/D_2O, H_2SO_4 and $KMnO_4$ by ^{17}O NMR: First, oxygen exchange between water and sulfuric acid was monitored by liquid NMR, and the following solutions were prepared (Figure S7A): a) water containing 4% ^{17}O-water by dilution from 40% ^{17}O-water; b) pure conc. H_2SO_4 (~98%, as obtained); c and d) pure conc. H_2SO_4 (~94%), diluted by addition of a calculated amount of ^{17}O-water (40%) after 5 and 31 minutes, respectively. ^{17}O ssNMR (67.8 MHz, **Figure S7A**): δ -2.6 – 15.4 (H_2O/H_3O^+), δ 149.3 (H_2SO_4).

In another set of experiments, the oxygen-isotope exchange between the acid mixture and potassium permanganate was monitored by liquid NMR (Figure S7B) as follows: a) $KMnO_4$ was dissolved in D_2O at a concentration of 0.25 M and measured after 24 hours at room temperature; b and c) $KMnO_4$ was dissolved in a mixture of D_2O (800 µl) with ^{17}O-water (40%, 200 µl) at a final concentration of 0.25 M. the obtained solution as subsequently mixed with 1000 µl of 0.05 M HCl; d) KMnO4 (52.4 mg) was dissolved in 1 ml H_2SO_4 and stirred for at least 1 hour. The mixture reflects approximately the ratio of reagents used for the oxidation of graphite. After 30 minutes, 4 hours and 24 hours, a small amount of the mixture (100 µl) was diluted with D_2O and ^{17}O-spectra were recorded within 5 minutes. All three spectra were almost identical. ^{17}O ssNMR (67.8 MHz, **Figure S7B**): δ -3.0 – 1.0 (H_2O/H_3O^+), δ 157.5 (H_2SO_4), δ 1210.9-1209.2 ($KMnO_4$).

Hydrolysis of organosulfates: A freshly synthesized batch of GO was prepared according the general protocol of synthesis. Directly after synthesis, one share was of the material was freeze-dried overnight to determine the concentration of the material. The stock dispersion was adjusted to 0.6 mg/mL. After certain time intervals, a sample was centrifuged at 12.000 RCF for 30 minutes. The supernatant was analyzed by ion chromatography and the conductivity was determined. A set of 14mL deposable centrifugation tubes was filled with each 10 mL of the dispersion. One set of five was stored at 4°C in the refrigerator, another set of five stored at ambient conditions (25°C) in a locker. Two other sets of five were placed into a water bath with 40°C and 60°C, respectively. The same was done for the zero-sample at the start of the experiment directly after adjustment of the concentration (**Figure 4D**). For hydrolyzation at elevated temperatures over the course of several days, two almost identical GO dispersions were used (**Figure 4E**). One pair of 5 was incubated at 40°C and 60°C, the other pair was used at 60°C and 80°C. EA of GO-1: C = 42.50 %; H = 2.63 %; N < 0.5%; S = 3.91 %. GO-2: C = 38.90 %; H = 2.52 %; N < 0.5%; S = 4.97.

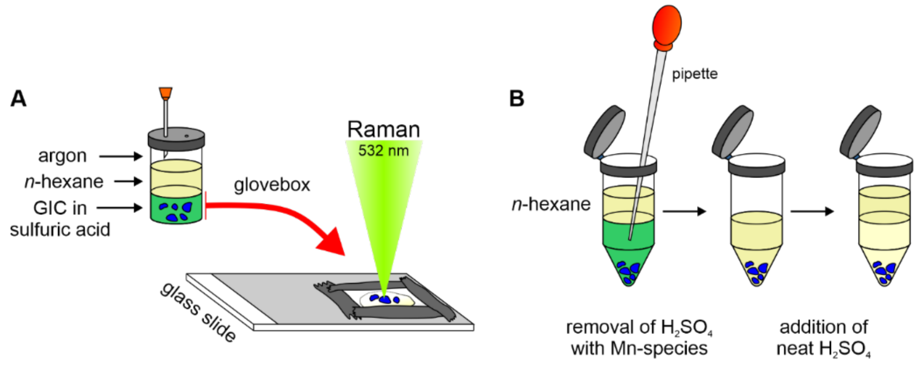

Figure S1: *(A) Setup for in situ oxidation and transfer of prepared graphite intercalation compounds (GICs) for Raman spectroscopy. (B) Washing procedure of GIC^{PM} outside of the glove box with n-hexane at ambient conditions.*

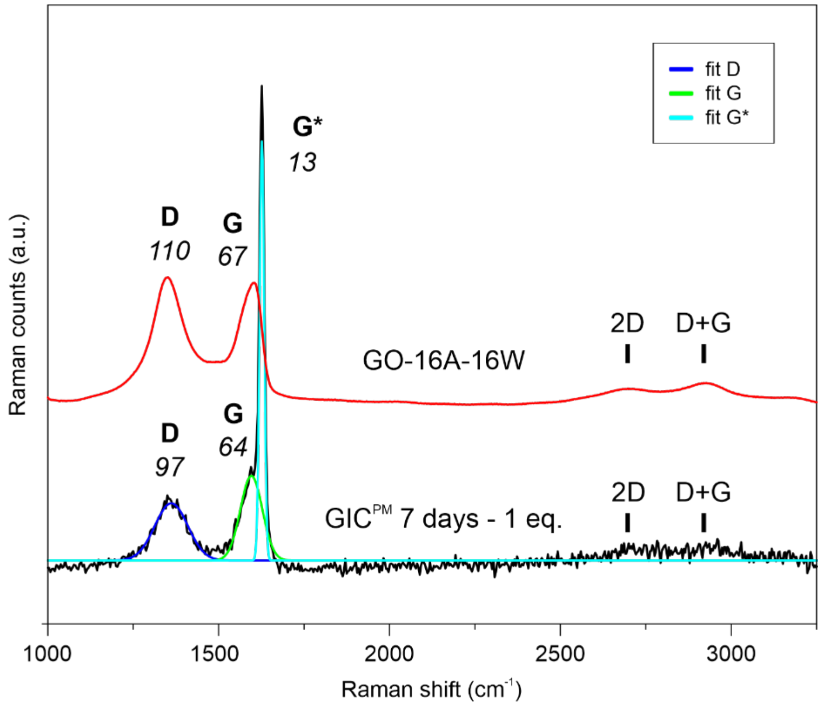

Figure S2*: Raman spectrum of GO-16A-16W and GIC^{PM} after 7 days in the reaction mixture with one weight equivalent of potassium permanganate and GO on 300 nm SiO_2/Si wafer. The D and G Raman mode of functionalised graphene layers are indicated with letters at the corresponding positions, while G* indicates the Raman mode of stage-1 intercalated layers. The corresponding full-width-at-half-maximum of fitted Gauss-functions are given in italic numbers.*

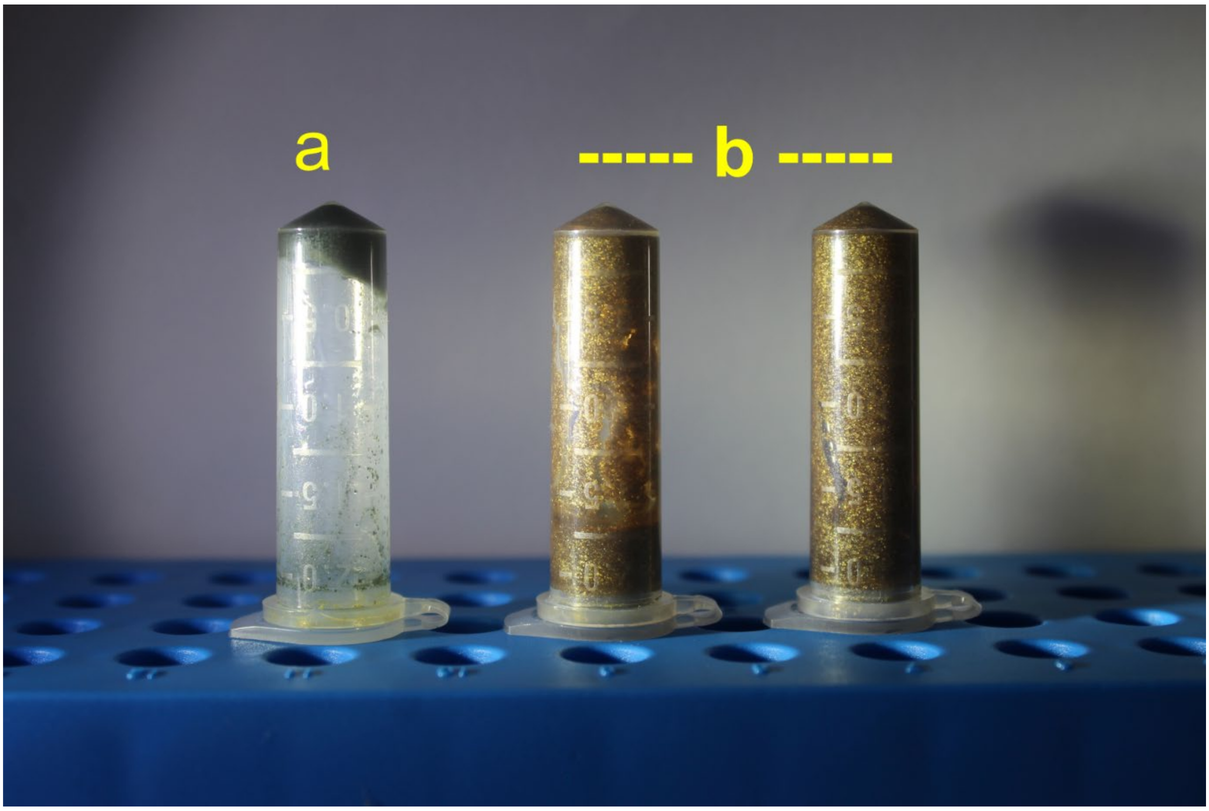

Figure S3*: Photograph of oxidized graphite crystals with permanganate before (a) and after addition of water and hydrogen peroxide solution (b).*

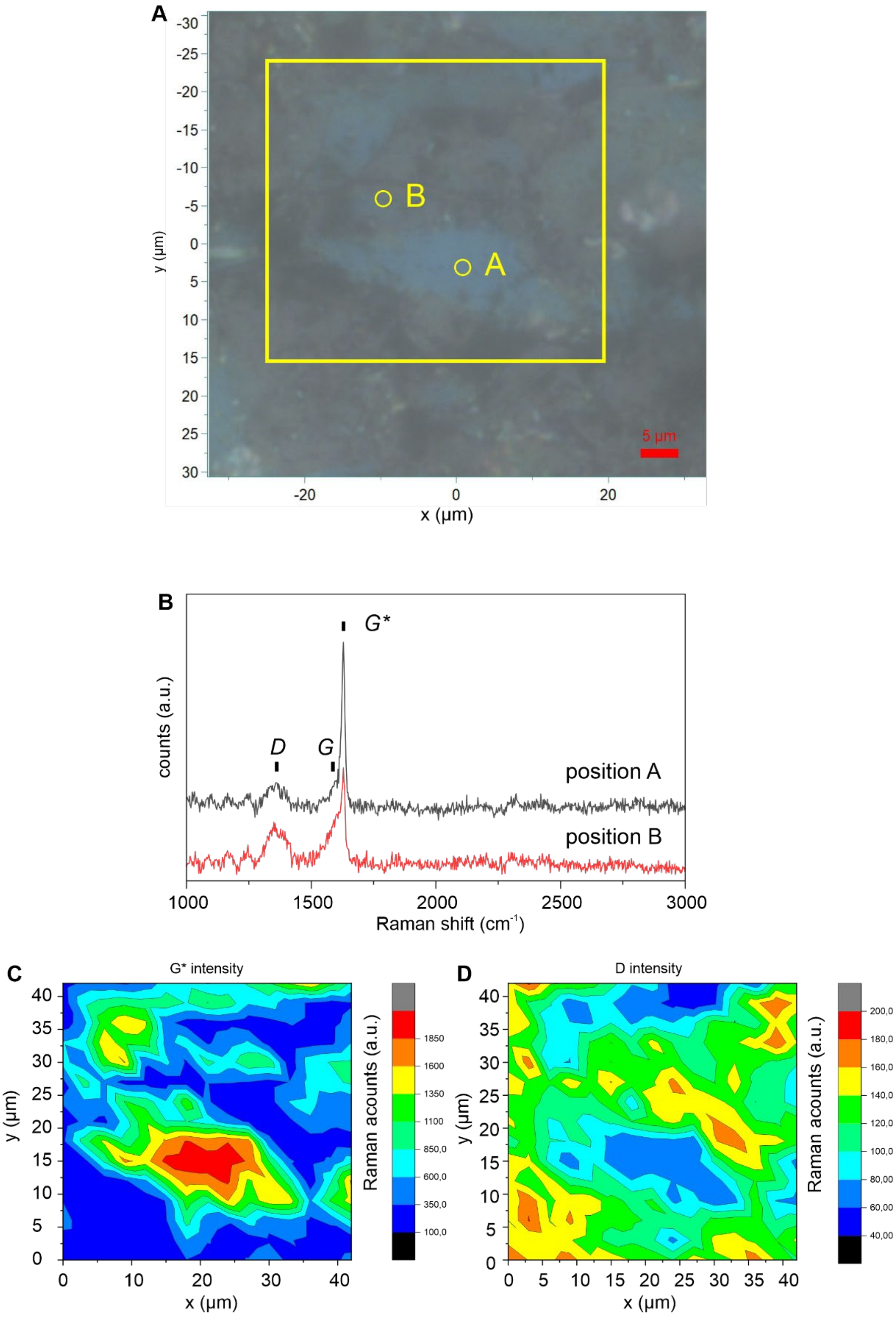

Figure S4: *(A) Overview of the surface of a graphite particle, oxidized for 7 days in sulfuric acid with potassium permanganate. (B) Raman spectra of two different spots on the graphite surface. A higher intensity of G* stemming stage-1 intercalated domains can be observed in the blueish areas. (C, D) Heat maps of a selected area of the particle surface with respect to the G* and D mode intensity.*

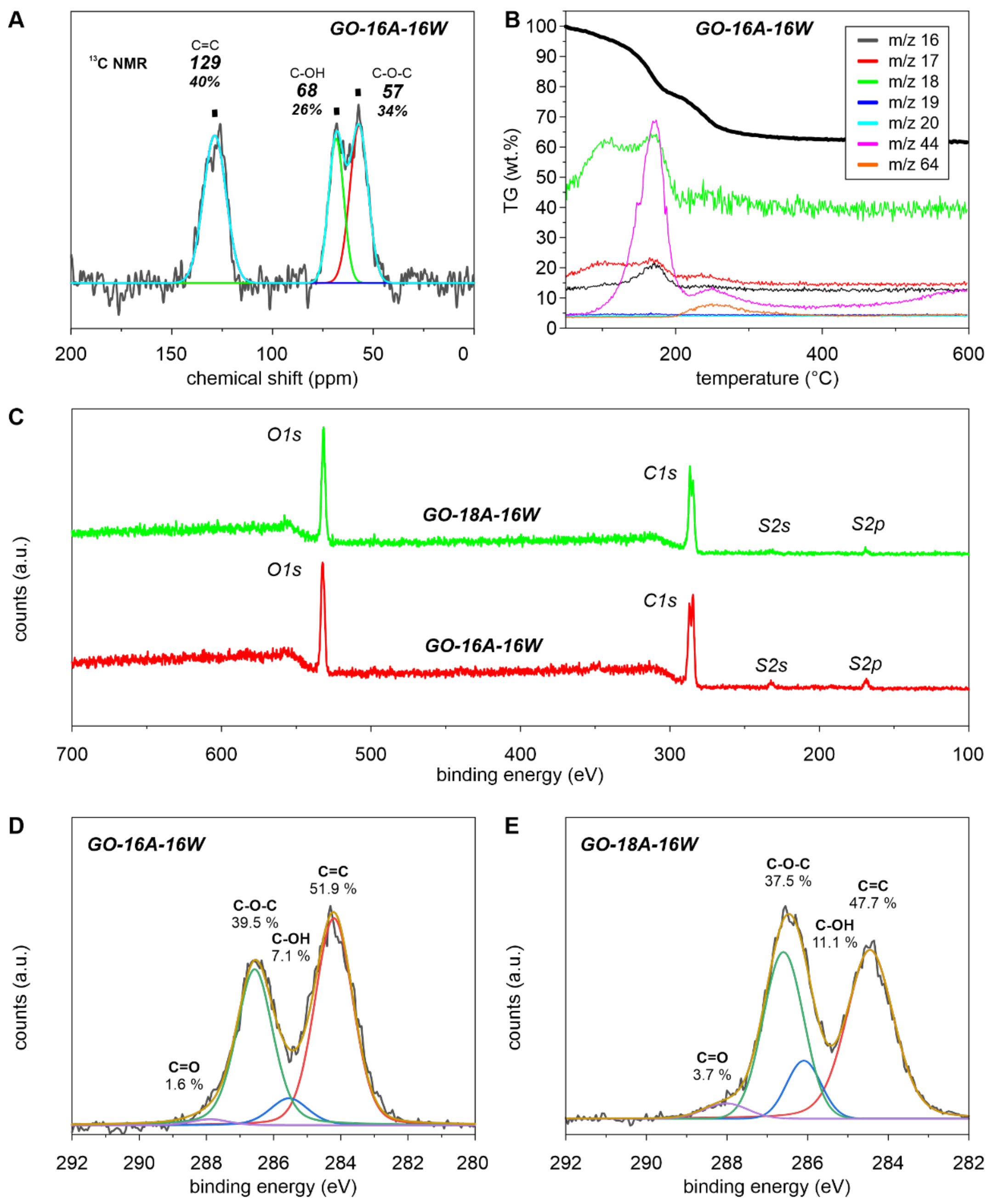

Figure S5: *(A) Solid state NMR of GO-16A-16W. The corresponding functional groups are annotated together with their corresponding shift in bolt letters and the percentage of the integrated area in italic. (B) TGA-MS profile of GO-16A-16W. The corresponding m/z values of the cleaved fragments are shown in the inlet. (C) XPS survey spectra of GO-16A-16W and GO-18A-16W on gold substrate. The corresponding C1s high resolution spectra are shown in (D) for and (E). The functional groups are annotated together with their corresponding fraction in percent below.*

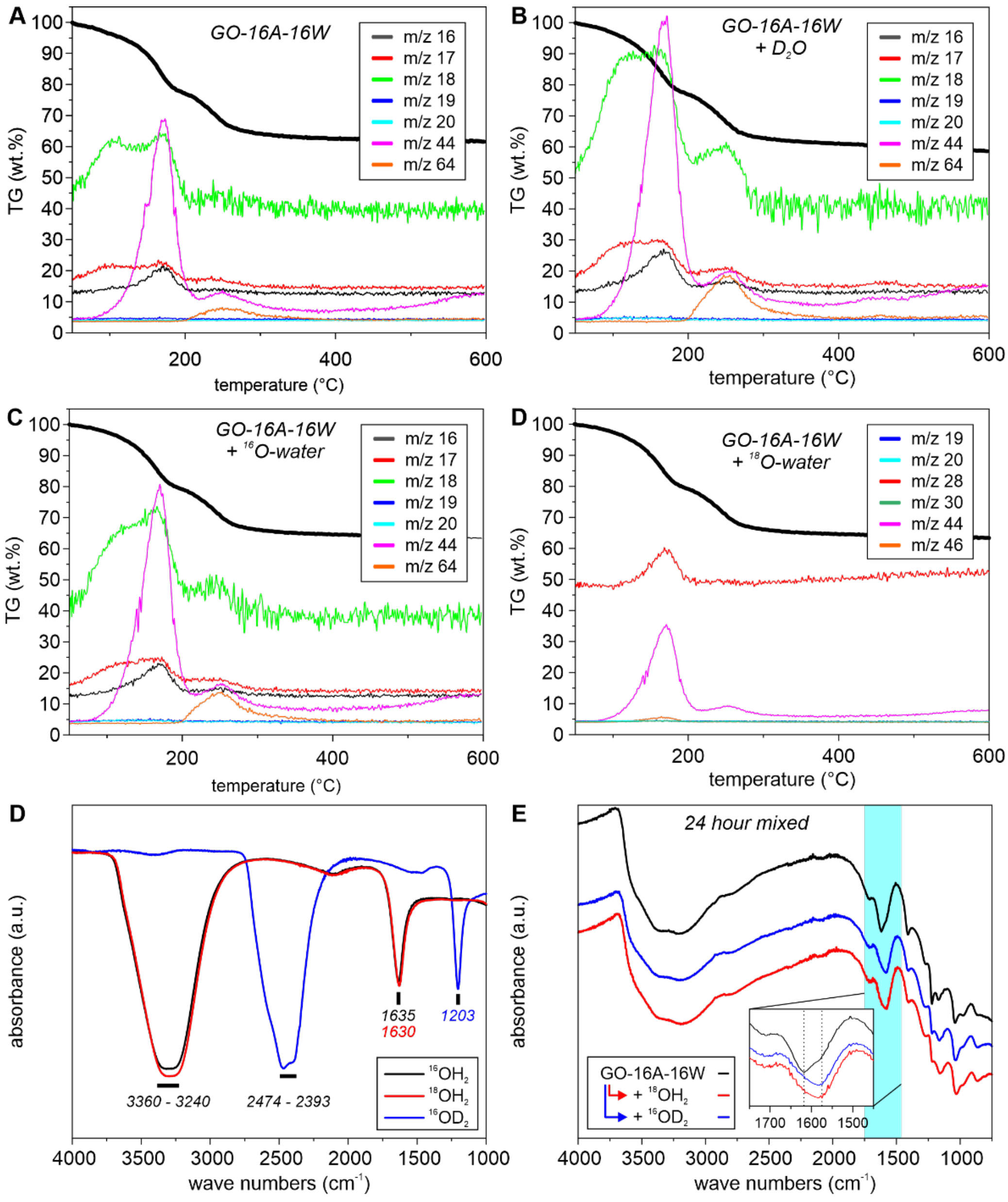

Figure S6: *TGA-MS spectra of the initial GO-16A-16W after incubation with (A) DI-water, (B) D_2O, or (C) ^{18}O-labelled water for few hours. The samples were subsequently freeze driend and used as obtained for analysis. (D) Same TGA-MS spectrum as (C) but with a focus on m/z values for OH and OH_2 (m/z 19, 20), labelled and unlabelled CO (m/z = 28, 30) and CO_2 (m/z = 44, 46). (D) FTIR spectra of DI water, ^{18}O-labveleld water and D^2O. (E) FTIR spectra of GO before and after incubation with ^{18}O-water or D_2O for 24 hours. The inlet represents a magnifictation of the spectral range, where C=C double bonds (~1575 cm^{-1}) and vibration from adsiprbed water (~ 1620 cm^{-1}) are visible. For D_2O, no new vibrational bands are arising with respect to the spectrum of initial GO. Similarly, no shift in the band at 1620 cm^{-1} can be observed after incubation with ^{18}O-water.*

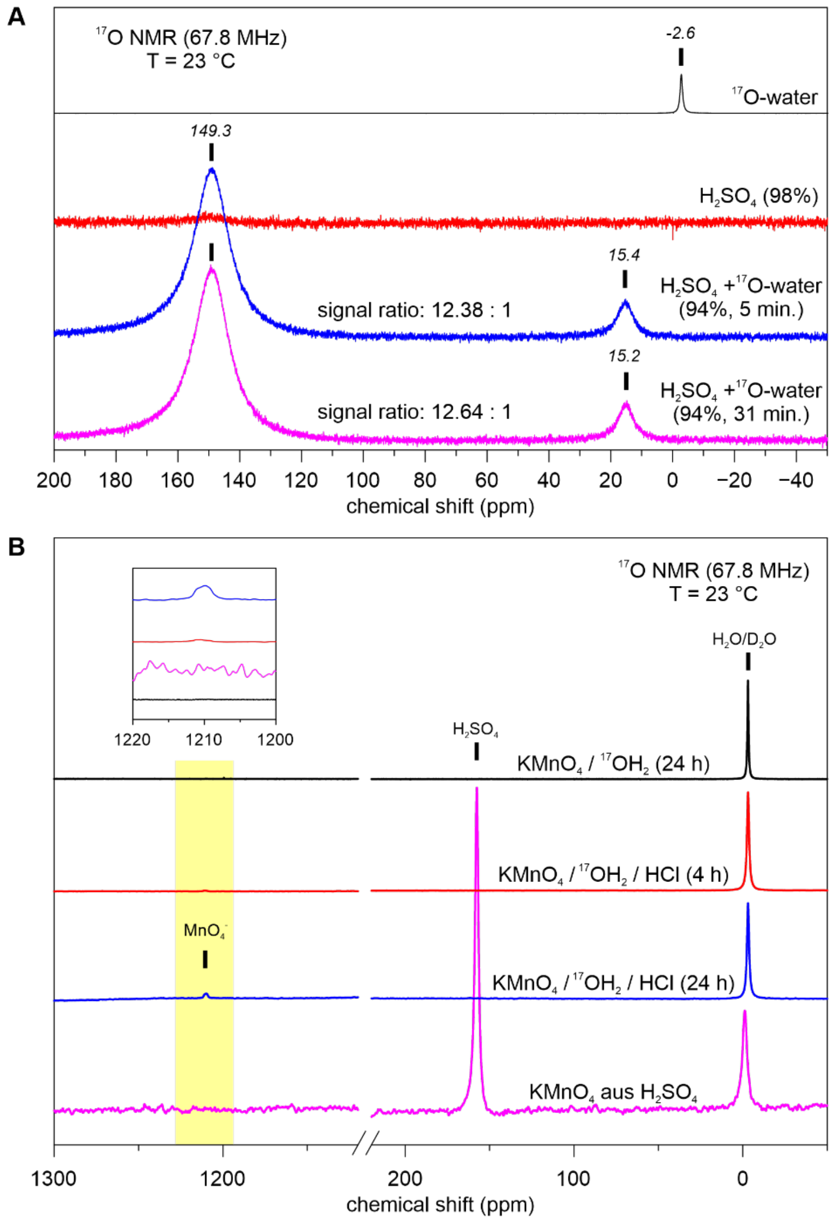

Figure S7: *^{17}O-NMR reference spectra to monitor possible oxygen-echange bewen the water, sulfuric acid and potassium permanganate. (A) water cointaining 4% ^{17}O (black), pure sulfuric acid (~98%, red), and sulfuric acid slightly diluted with ^{17}O-water water after 5 minutes (blue) and 31 minutes (purple). The signal ratio reflects the equilibrium of ~94 % sulfuric acid. (B) ^{17}O-NMR spectrum of 0.25 M $KMnO_4$ in D_2O after 24 hours (black), 0.125 M KMnO4 containing 4% of ^{17}O-water and acidified with hydrochloric acid after 4 hours (red) and 24 hours of incubation (blue), and $KMnO_4$ recovered from concentrated sulfuric by dilution of 100 µl of the acid mixture with 900 µl D_2O (purple). As it can be seen in the inlet, after 24 hours of reaction time, a new signal at around 1210 ppm stemming from ^{17}O-$KMnO_4$ appears in the acidified aqueous solution of $KMnO_4$, which is absent for the other samples* [2].

(1) Holm, A.; Wrasman, C. J.; Kao, K. C.; Riscoe, A. R.; Cargnello, M.; Frank, C. W. Langmuir-Blodgett Deposition of Graphene Oxide-Identifying Marangoni Flow as a Process that Fundamentally Limits Deposition Control. *Langmuir* **2018**, *34* (33), 9683-9691. DOI: 10.1021/acs.langmuir.8b00777.
(2) Tarasov, V. P.; Kirakosyan, G. A. 16O/18O oxygen isotope exchange kinetics in MnO_4 − as probed by 55Mn NMR. *Russ. J. Phys. Chem. B* **2016**, *10* (4), 582-586. DOI: 10.1134/s1990793116040278.
